# Study of Grape Polyphenols by Liquid Chromatography-High-Resolution Mass Spectrometry (UHPLC/QTOF) and Suspect Screening Analysis

**DOI:** 10.1155/2015/350259

**Published:** 2015-02-04

**Authors:** Riccardo Flamini, Mirko De Rosso, Luigi Bavaresco

**Affiliations:** ^1^Consiglio per la Ricerca e la Sperimentazione in Agricoltura, Centro di Ricerca per la Viticoltura (CRA-VIT), Laboratorio Chimico, Viale XXVIII Aprile 26, 31015 Conegliano, Italy; ^2^Istituto di Frutti-Viticoltura, Università Cattolica S.C., Via Emilia Parmense 84, 29122 Piacenza, Italy

## Abstract

Suspect screening analysis is a targeted metabolomics method in which the identification of compounds relies on specific available information, such as their molecular formula and isotopic pattern. This method, coupled to liquid chromatography-high-resolution mass spectrometry, is effective in the study of grape metabolomics, in particular for characterization of flavonols, stilbene derivatives, and anthocyanins. For identification of compounds expected in the samples, a new database of putative compounds was expressly constructed by using the molecular information on potential metabolites of grape and wine from the literature and other electronic databases. Currently, this database contains around 1,100 compounds. The method allows identification of several hundred grape metabolites with two analyses (positive and negative ionization modes), and performing of data reprocessing using “untargeted” algorithms also provided the identification of some flavonols and resveratrol trimers and tetramers in grape for the first time. This approach can be potentially used in the study of metabolomics of varieties of other plant species.

## 1. Introduction

Polyphenols are the principal grape bioactive compounds, which are characterized by antioxidant and peroxyl radical scavenger activity, form complexes with metals (Cu, Fe, etc.), and show ability to cross the mammal intestinal wall ([1] and references cited herein).

Flavonols are among the principal classes of bioactive polyphenols and are mainly present in the berry skin. Principals are quercetin, kaempferol and myricetin 3-*O*-glucoside, and 3-*O*-glucuronide and rutin. Recently, also isorhamnetin, laricitrin and syringetin 3-*O*-glucoside, kaempferol and laricitrin 3-*O*-galactoside, kaempferol-3-*O*-glucuronide, and quercetin and syringetin 3-*O*-(6-*O*-acetyl)glucoside were found in grape [2, 3]. Quercetin (the main grape flavonol) showed to block the human platelets aggregation and inhibits the cancer cell growth in human tumors ([4] and references cited herein).

Anthocyanins are present in the berry skin and are responsible of red color of grape and wine. In* V. vinifera* grapes, the principals are delphinidin (Dp), cyanidin (Cy), petunidin (Pt), peonidin (Pn) and malvidin (Mv) 3-*O*-monoglucoside (also pelargonidin 3-*O*-monoglucoside was found in grape [5]), 3-*O*-acetylmonoglucoside, 3-*O*-(6-*O*-*p*-coumaroyl)monoglucoside, and Mv-3-*O*-(6-*O*-caffeoyl)monoglucoside. Non-*V. vinifera* red grape varieties (hybrid) often also contain diglucoside anthocyanins with a second glucose molecule linked to C-5 hydroxyl group.

Grape anthocyanins are studied for oenological purposes and because they are antioxidant compounds and natural colorants used in nutraceutical, food, and pharmaceutical industry [6–8]. Moreover, they are also studied for chemotaxonomic purposes: for example, the presence of 3,5-*O*-diglucoside is a marker to distinguish between* V. vinifera* and hybrid grapes, the former being characterized by lower presence, or the absence, of these compounds [9].

Another important class of grape polyphenols is the stilbene derivatives, such as* cis*- and* trans*-resveratrol (3,5,4′-trihydroxystilbene), resveratrol glucoside (*Z* and* E* piceid), resveratrol dimers (viniferins) and oligomers, piceatannol (3,4,3′,5′-tetrahydroxy-*trans*-stilbene), and piceatannol glucoside (*E* and* Z* astringin) [11]. In general stilbenes act as phytoalexins in soft tissues, while grapevine synthesizes *ε*-viniferin, *δ*-viniferin glucoside, and pallidol in roots, cluster steams, and stems as constitutive compounds [12, 13]; viniferins can also form in grape tissues by oligomerization of* trans*-resveratrol as active defense against exogenous attacks or be produced from resveratrol by extracellular enzymes released from the pathogen in an attempt to eliminate undesirable toxic compounds [14, 15]. Also* E* and* Zω*-viniferin and some resveratrol trimers and tetramers, such as ampelopsin D, quadrangularin A, *α*-viniferin,* E*- and* Z*-miyabenol C, isohopeaphenol, ampelopsin H, and vaticanol C-like, were found in grapevine leaves, roots, clusters, and stems [16], and* E*-ampelopsin E,* E*-amurensin B,* E*-resveratroloside,* E*-3,5,4′-trihydroxystilbene 2-C-glucoside,* Z*-ampelopsin E, scirpusin A, and* E*- and* Z*-vitisin B were found in grapevine canes [17].

Several* in vitro* studies demonstrated the anticancer, antioxidant, anti-inflammatory, cardioprotective, and platelet aggregation inhibition activity of resveratrol [18–24]. Piceatannol blocks LMP2A, a viral protein-tyrosine kinase implicated in leukemia, non-Hodgkin's lymphoma, and other diseases associated with the Epstein-Barr virus (EBV) [25, 26] and showed to act on human melanoma cells [27].

Analytical methods commonly used to study grape polyphenols are performed by reverse-phase high performance-liquid chromatography (HPLC) coupled with spectrophotometry or mass spectrometry (MS) [28, 29]. In general, liquid chromatography/mass spectrometry (LC/MS) and multiple mass spectrometry (MS/MS and MS^*n*^) are the more effective tool for the structural characterization of these low-molecular weight (MW) compounds. LC/MS of flavonols in grape extracts was performed in both positive and negative ionization modes [2, 10], and analysis of resveratrol and piceatannol is usually performed in negative mode [30, 31] and structural characterization of anthocyanins in positive LC/MS^*n*^ [32]. Due to the lack of standards commercially available, identification of compounds is also confirmed on the basis of their elution sequence from the column. Moreover, fast methods by direct injection and positive electrospray ionization and multiple mass spectrometry (ESI-MS/MS) for structural characterization and semiquantitative profiling on the anthocyanins in grape extracts were developed [33, 34].

Collision-induced-dissociation (CID) by using He as collisional gas is highly effective in differentiation of isobaric anthocyanins: the fragment ions [M-162]^+^, [M-324]^+^ (formed by two consecutive losses of sugar residue), [M-204]^+^, [M-308]^+^, and [M-470]^+^ (consecutive losses of* p*-coumaroylglucose and glucose) characterize both the monoglucoside and diglucoside compounds. Only in the case of Mv-3,5-*O*-diglucoside and Mv-3-*O*-(6-caffeoyl)monoglucoside, which have the same nominal mass and aglycone, MS^*n*^ is not able to distinguish the two compounds.

“Metabolomics” should provide the comprehensive quantitative and qualitative study of all these grape metabolites. In general, “untargeted metabolomics” methods have high sensitivity, good resolution, and high-throughput capacity and should be able to reveal a great number of candidate biomarker signals in a single run [35]. On the other hand, “targeted metabolomics” is used for the quantitative study of specific compounds but with limited information on the sample metabolome [36, 37].

*Suspect screening analysis* is a midway approach. The identification method relies on the availability of specific information on the metabolites in the sample—by using high-resolution MS—in particular, their molecular formula and structure [38]. This approach has been applied to the study of grape metabolomics, and a new database (*GrapeMetabolomics*) has been constructed by including the information on grape and wine metabolites found in the literature and others in electronic databases and the new compounds identified by “untargeted metabolomics” in the samples studied until now. Currently, this database contains more than 1,100 grape and wine putative metabolites with molecular weight between 100 and 1700 Da [39].

This paper describes the study of grape flavonols, anthocyanins, and stilbenes performed by ultrahigh performance-liquid chromatography/quadrupole-time of flight mass spectrometry (UHPLC/QTOF) and* suspect screening* metabolomics.

## 2. Materials and Methods

### 2.1. Chemicals and Sample Preparation

Standards of quercetin, myricetin, kaempferol, quercetin glucoside, malvidin-3-*O*-glucoside, malvidin-3,5-*O*-diglucoside, and rutin were purchased from Extrasynthese (Genay, France);* trans*-resveratrol, piceatannol,* E*-piceid, and 4′,5,7-trihydroxy flavanone were purchased from Sigma-Aldrich (Milan, Italy) and* Z*-piceid was produced by photoisomerization of* E* isomer with a conversion yield of 83% by using the same conditions reported for isomerization of* trans*-resveratrol [28].

Thirty-four hybrid grape varieties (22 red and 12 white obtained by crossing* V. vinifera*,* V. riparia*,* V. labrusca*,* V. lincecumii*, and* V. rupestris*) and 10* V. vinifera* grape varieties (Corvina, Enantio, Lambrusco Grasparossa, Montepulciano, Nebbiolo, Nero d'Avola, Primitivo, Raboso Piave, Rossese, and Uva di Troia) were harvested in 2010 and 2012, respectively, at full ripeness (maximum sugar content) from five plants present in the CRA-VIT grapevine germplasm repository (Susegana, Veneto, Italy). For each sample, about 100 berries were picked randomly and immediately frozen at −20°C.

For sample preparation, twenty berries were weighed and homogenized using liquid nitrogen and the resulting powder was immediately extracted with pure methanol in ratio 2 : 1 v/w under stirring for 20 min in the dark. After addition of 200 *μ*L of internal standard 4′,5,7-trihydroxy flavanone 500 mg/L, the sample was centrifuged at 10°C for 20 min by keeping the sample away from light by wrapping with aluminum foil. The solution was filtered with Acrodisc GHP 0.22 *μ*m filter (Waters) and collected in a vial for LC/MS analysis.

### 2.2. LC/QTOF Mass Spectrometry

The analytical system used was Agilent UHPLC 1290 Infinity coupled to Agilent 1290 Infinity Autosampler (G4226A) and Agilent 6540 accurate-mass Q-TOF mass spectrometer (nominal resolution 40.000) with Jet Stream Ionization source (Agilent Technologies, Santa Clara, CA). Two analyses of each sample were repeated by recording full scan acquisition in both positive and negative ionization modes. After each sample a blank was run. The data acquisition software was Agilent MassHunter version B.04.00 (B4033.2). Chromatography was performed using a Zorbax reverse-phase column (RRHD SB-C18 3 × 150 mm, 1.8 *μ*m) (Agilent Technologies, Santa Clara, CA). The mobile phase was composed of (A) 0.1% v/v aqueous formic acid and (B) 0.1% v/v formic acid in acetonitrile. Gradient elution program: 5% B isocratic for 8 min, from 5% to 45% B in 10 min, from 45% to 65% B in 5 min, from 65% to 90% in 4 min, and 90% B isocratic for 10 min. Flow rate was 0.4 mL/min; sample injection was 10 *μ*L; column temperature was 35°C.

QTOF conditions are as follows: sheath gas nitrogen 10 L/min at 400°C, drying gas 8 L/min at 350°C, nebulizer pressure 60 psig, nozzle voltage 1 kV, and capillary voltage 3.5 kV. Signals in the* m/z* 100–1700 range were recorded. Negative mass calibration was performed with standard mix G1969-85000 (Supelco Inc.) and had residual error for the expected masses between ±0.2 ppm. Lock masses are as follows: TFA anion at* m/z* 112.9856 and HP-0921(+formate) at* m/z* 966.0007 in negative-ion mode and purine at* m/z* 121.0509 and HP-0921 at* m/z* 922.0098 in positive-ion mode. MS/MS conditions are as follows: collision energy between 20 and 60 eV was used to fragment ions in the* m/z* 100–1700 range. Acquisition rate was 2 spectra/s.

### 2.3. Data Analysis

Data processing was performed with Agilent MassHunter Qualitative Analysis software version B.05.00 (5.0.519.0). The database of putative grape and wine metabolites (*GrapeMetabolomics*) was constructed by including the information on their molecular formulae from the literature and found in electronic databases. “Targeted” data processing was performed by using the algorithm “Find Compounds by Formula” and “untargeted” data reprocessing by the algorithm “Find Compounds by Molecular Feature.”

Confidence of the compound identification was based on accurate mass and isotope pattern and was expressed by an “overall identification score” computed as a weighted average of the compound isotopic pattern signals, such as exact masses, relative abundances, and* m/z* distances (spacing). Weight parameters were *W*_mass_ = 100, *W*_abundance_ = 60, and *W*_spacing_ = 50; mass expected data variation 2.0 mDa + 5.6 ppm, mass isotope abundance 7.5%, and mass isotope grouping peak spacing tolerance 0.0025* m/z* + 7.0 ppm.

## 3. Results and Discussion

### 3.1. High-Resolution MS and Suspect Screening Analysis

To limit artifacts and possibility of false negative grape extract was prepared by operating in cold condition and with a minimal sample handling. To avoid false positives, a blank was run after each sample and the background was subtracted to the sample chromatogram.

In high-resolution mass spectrometry (HRMS) the compounds are identified by performing the raw data processing using specific algorithms. In this method, molecular formulae (MFs) are calculated by recording the monoisotopic mass and isotopic pattern of the compounds (relative abundances and* m/z* distances of the signals) [40, 41]. Compounds are then identified by searching in the databases of metabolites available [42–44]. Unfortunately, databases specific for grape do not exist and to overcome this problem a method of suspect screening analysis was developed. MFs are calculated by using “targeted” algorithms, which perform deconvolution of the chromatogram and the comparison between the theoretical and experimental isotopic patterns, and the compounds expected in the sample are identified by using a homemade database of putative grape and wine metabolites (*GrapeMetabolomics*).

After targeted search, the raw data reprocessing by using “untargeted” algorithms provides a list of MF not present in* GrapeMetabolomics* or with retention times (RT) different from those in the database. When the search in other databases provides the identification of new compounds, with high confident score and compatible with the sample, they are added to the database with the correspondent RT. As a consequence, by increasing the number of samples studied the database itself can be expanded. Identifications are confirmed by multiple mass spectrometry (MS/MS) and accurate mass measurement of fragments [39]. In general, by performing two analyses in positive and negative ionization modes, about 320–450 putative compounds are identified in a grape extract with overall identification score higher than 60%, including anthocyanins, flavones and flavanones, flavanols and procyanidins, stilbenes, phenolic acids, and glycoside aroma precursors, about 30–60 compounds with identification score higher than 99%, and more than hundred higher than 95%. In general, identification score can be affected by low signal intensity (which mainly influenced the score parameters of isotopic patterns) and matrix background in the chromatogram [38]. Since standards of most compounds are not available, identification is performed by overlapping various analytical approaches (accurate mass and isotopic pattern, MS/MS fragmentation, correlation between fragments observed, and putative structure) in agreement with the indications recommended in MS-based metabolomics [45].

### 3.2. Flavonols

**Figure 1:**
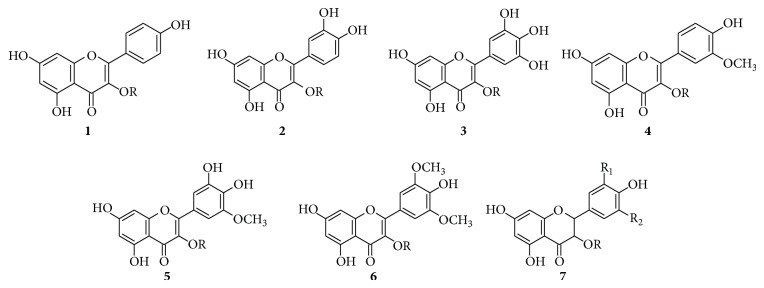
Main flavonols in grape. R: galactose, glucose, glucuronic acid, and rutinose; R_1_: H, OH, and OCH_3_; R_2_: H, OH, and OCH_3_;** (1)** kaempferol,** (2)** quercetin,** (3)** myricetin,** (4)** isorhamnetin,** (5)** laricitrin,** (6)** syringetin, and** (7)** dihydroflavonol.

The structures of main flavonols identified in* V. vinifera* grapes are shown in Figure 1. By performing analysis in negative-ion mode, twenty-four glycoside flavonols were identified in some hybrid grape samples (Seibel 19881 and Seyve Villard 12-347), including 4 quercetin, 5 myricetin, 4 kaempferol, 3 isorhamnetin, 2 laricitrin and 3 syringetin derivatives, and 3 dihydroflavonols [10].

**Figure 2:**
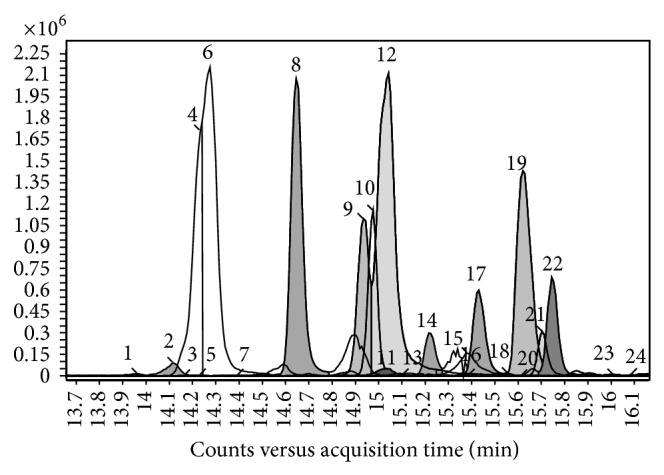
Overlapping of the extracted ion chromatograms (EIC) of [M-H]^−^ ion signals of glycoside flavonols identified in UHPLC/QTOF analysis of hybrid grape Seyve Villard 12-347.

Figure 2 shows the overlapped extracted ion chromatograms (EIC) of the [M-H]^−^ ion signals recorded in the analysis of Seyve Villard 12-347 sample. Compounds identified are listed in Table 1 with their experimental [M-H]^−^ ions.

Flavonols were identified with an error mass lower than 3 ppm and with identification score higher than 90%. Lower overall identification scores were found for kaempferol galactoside, kaempferol glucuronide, and dihydrokaempferol rhamnoside, probably due to the low signals intensity [39].

Four compounds were identified in grape for the first time: two myricetin derivatives with nominal mass at* m/z* 656 and* m/z* 642, respectively, a syringetin derivative at* m/z* 670 and an isorhamnetin derivative at* m/z* 624. Identification was confirmed by accurate MS/MS: all compounds showed the aglycone fragment and a aglycone-sugar fragment was observed for some of them. New glycoside flavonols identified were myricetin hexoside-glucuronide, a myricetin* O*-di-hexoside, a syringetin* O*-di-hexoside, and isorhamnetin rutinoside.

### 3.3. Anthocyanins

**Figure 3:**
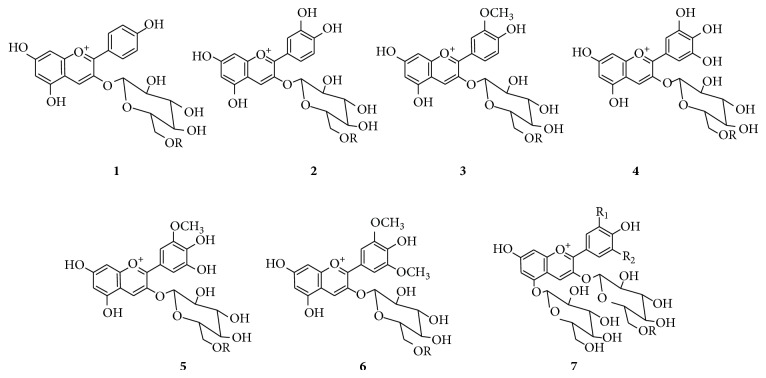
Principal grape anthocyanins. R: H, acetyl,* p*-coumaroyl, and caffeoyl; R_1_: H, OH, and OCH_3_; R_2_: H, OH, and OCH_3_;** (1)** pelargonidin-3-*O*-glucoside,** (2)** cyanidin-3-*O*-glucoside,** (3)** peonidin-3-*O*-glucoside,** (4)** delphinidin-3-*O*-glucoside,** (5)** petunidin-3-*O*-glucoside,** (6)** malvidin-3-*O*-glucoside, and** (7)** anthocyanidin-3,5-*O*-diglucoside.

**Figure 4:**
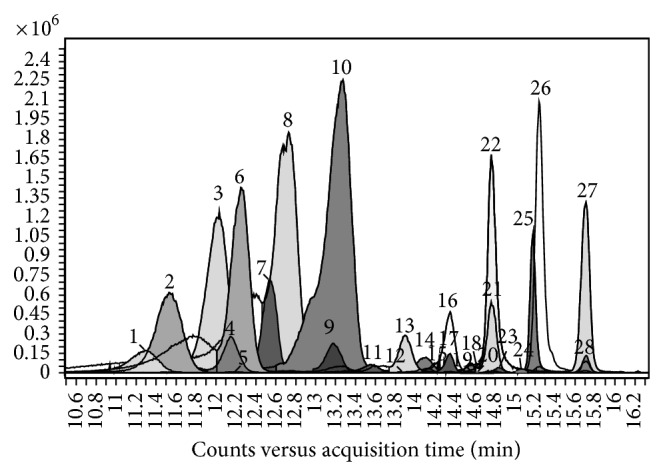
Extracted ion chromatograms of M^+•^ ion signals of anthocyanins identified in the UHPLC/QTOF analysis of Clinton grape extract.

Figure 3 shows the structures of principal anthocyanins identified in* V. vinifera* grapes. Highly selective and sensitive analysis performed in positive ionization mode allowed the identification of twenty-eight different anthocyanins in some hybrid grapes.  Figure 4 shows the overlapped extracted ion chromatograms of M^+•^ anthocyanin signals recorded in the analysis of Clinton grape (*V. labrusca* ×* V. riparia*). Compounds identified are listed in Table 2.

If some isobaric anthocyanins overlap in the chromatogram they can be identified by using a MS system of at least 30.000 resolution. In particular, Cy-3,5-*O*-diglucoside (C_27_H_31_O_16_, MW 611.1612) and Dp-3-*O*-(6-*O*-*p*-coumaroyl)monoglucoside (C_30_H_27_O_14_, MW 611.1401), Pn-3,5-*O*-diglucoside (C_28_H_33_O_16_, MW 625.1769) and Pt-3-*O*-(6-*O*-*p*-coumaroyl)monoglucoside (C_31_H_29_O_14_, MW 625.1557), Mv-3,5-*O*-diglucoside (C_29_H_35_O_17_, MW 655.1874), and Mv-3-*O*-(6-*O*-caffeoyl)monoglucoside (C_32_H_31_O_15_, MW 655.1663) can be present in grape.

### 3.4. Stilbenes

**Figure 5:**
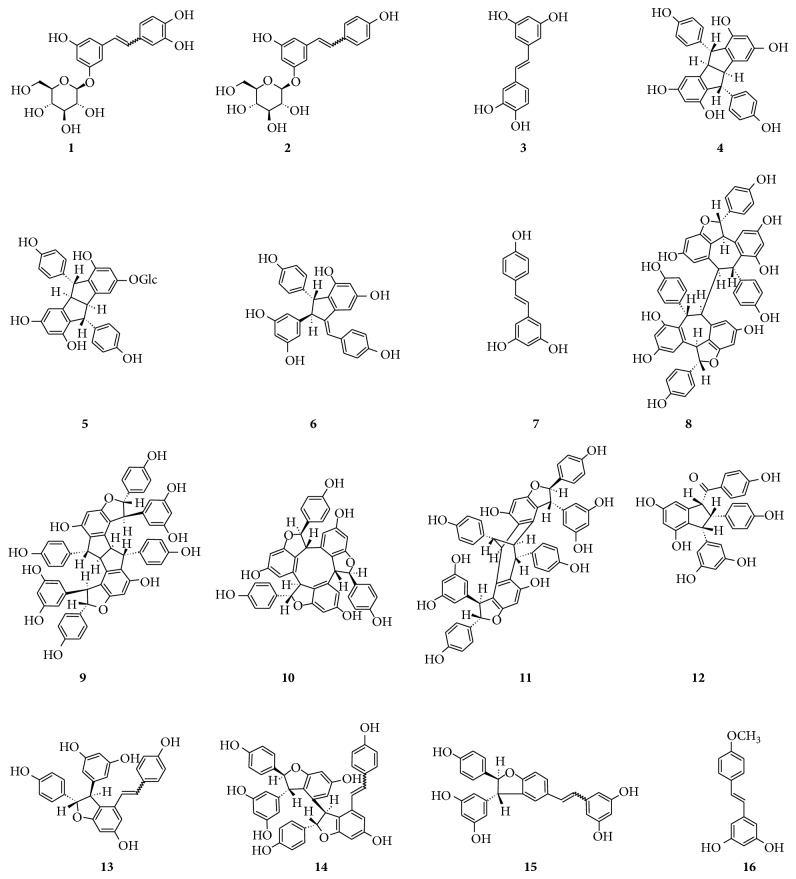
Principal stilbenes identified in vine tissues. (**1**)* E*- and* Z*-astringin, (**2**)* E*- and* Z*-piceid, (**3**) piceatannol, (**4**) pallidol, (**5**) pallidol-3-*O*-glucoside, (**6**) parthenocissin A, (**7**)* trans*-resveratrol, (**8**) hopeaphenol, (**9**) ampelopsin H, (**10**)*α*-viniferin, (**11**) vaticanol C isomer, (**12**) caraphenol B, (**13**)* E-* and* Z*-*ε*-viniferin, (**14**)* E*- and* Z*-miyabenol C, (**15**)* E*- and* Z*-*δ*-viniferin, and (**16**)* trans*-resveratrol-4′-methyl ether.

**Figure 6:**
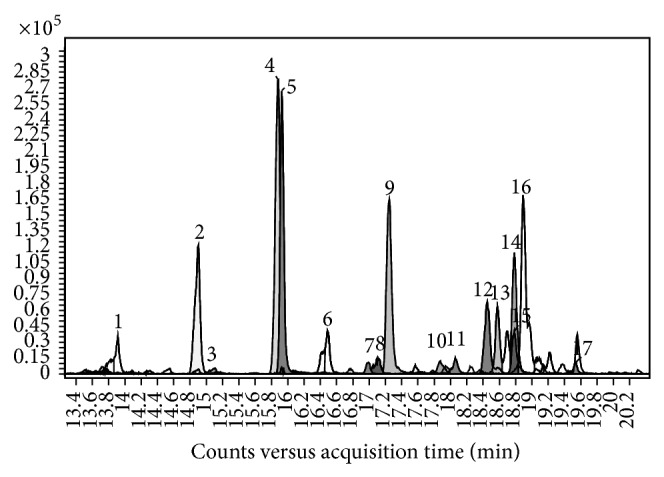
Extracted ion chromatograms of stilbene derivative [M-H]^−^ ion signals identified in UHPLC/QTOF analysis of Primitivo grape extract.

Principal stilbenes previously identified in vine tissues (leaves, roots, clusters, stems, and canes) are shown in Figure 5. By performing negative-ion analysis of Primitivo grape extract, seventeen stilbene derivatives were identified (Table 3) [39].  Figure 6 shows the EICs of their [M-H]^−^ signals.

The overall identification scores (ID scores) ranged between 98.1% (for a resveratrol dimer) and 99.8% (*cis*-piceid). Untargeted data processing allowed the identification of some resveratrol trimers and tetramers which were found in grape for the first time. Since several isomeric stilbene derivatives may be present in grape and MS cannot distinguish among them, tentative assignment was based on their column elution sequence and comparison with data from the literature [16, 46–59].

Precursor ions at* m/z* 679.197 were identified as* E*-miyabenol C and* Z*-miyabenol C, two resveratrol trimers previously found in* V. vinifera* leaves but not reported in grapes or wines [16, 52]. Also two resveratrol tetramers with precursor ions at* m/z* 905.260 were identified in grape for the first time: hopeaphenol (previously found in red wine [54]) and ampelopsin H/vaticanol C-like/isohopeaphenol (compounds previously found in* V. vinifera* leaves [16]).

## 4. Conclusions

High-resolution MS coupled to suspect screening analysis is effective in the study of grape metabolomics. With two analyses (positive and negative ionization modes) several hundred grape polyphenols are identified, and four flavonols and some grape resveratrol trimers and tetramers were found in grape for the first time.

Main difficulty of the method was the construction of the database specific for grape and wine which required relevant time and an accurate data collection. On the other hand, advantage of this method is that by increasing the studied samples the database can be expanded.

Despite the fact that* GrapeMetabolomics* is a database specific for the enological field a similar approach can be potentially applied to metabolomics of varieties of other plant species.

## Figures and Tables

**Table 1:** Glycoside flavonol [M-H]^−^ ions identified in hybrid grapes Seibel 19881 and Seyve Villard 12-347. Identification scores calculated with respect to the theoretical mass are reported. Numbers correspond to the peaks of chromatogram in Figure 2 (adapted from de Rosso et al., 2014 [10]).

Peak	Flavonols	Formula	Rt (min)	[M-H]^−^		Error (ppm)
				Experimental	Theoretical	
				*m*/*z*	*m*/*z*	
1	Syringetin *O*-di-hexoside	C_29_H_34_O_18_	14.00	669.1684	669.1672	1.7
2	Myricetin *O*-di-hexoside	C_27_H_30_O_18_	14.15	641.1373	641.1359	2.1
3	Myricetin hexoside-glucuronide	C_27_H_28_O_19_	14.16	655.1169	655.1152	2.5
4	Myricetin 3-*O*-galactoside	C_21_H_20_O_13_	14.26	479.0839	479.0831	2.0
5	Myricetin 3-*O*-glucuronide	C_21_H_18_O_14_	14.26	493.0635	493.0624	2.5
6	Myricetin 3-*O*-glucoside	C_21_H_20_O_13_	14.31	479.0844	479.0831	2.9
7	Dihydroquercetin 3-*O*-hexoside	C_21_H_22_O_12_	14.41	465.1046	465.1038	1.7
8	Rutin	C_27_H_30_O_16_	14.68	609.1471	609.1461	1.6
9	Quercetin 3-*O*-galactoside	C_21_H_20_O_12_	15.00	463.0894	463.0882	2.8
10	Quercetin 3-*O*-glucuronide	C_21_H_18_O_13_	15.02	477.0687	477.0675	2.6
11	Laricitrin 3-*O*-hexoside	C_22_H_22_O_13_	15.06	493.0998	493.0988	2.1
12	Quercetin 3-*O*-glucoside	C_21_H_20_O_12_	15.06	463.0895	463.0882	2.9
13	Laricitrin 3-*O*-glucuronide	C_22_H_20_O_14_	15.11	507.0789	507.0780	1.4
14	Kaempferol *O*-rhamnosyl-hexoside	C_27_H_30_O_15_	15.25	593.1518	593.1512	1.0
15	Dihydroquercetin 3-*O*-rhamnoside	C_21_H_22_O_11_	15.34	449.1097	449.1089	1.8
16	Isorhamnetin *O*-rhamnosyl-hexoside	C_28_H_32_O_16_	15.37	623.1603	623.1618	−2.4
17	Kaempferol 3-*O*-galactoside	C_21_H_20_O_11_	15.47	447.0938	447.0933	1.2
18	Syringetin 3-*O*-galactoside	C_23_H_24_O_13_	15.58	507.1147	507.1144	0.3
19	Kaempferol 3-*O*-glucoside	C_21_H_20_O_11_	15.65	447.0943	447.0933	2.3
20	Kaempferol 3-*O*-glucuronide	C_21_H_18_O_12_	15.66	461.0730	461.0725	1.0
21	Syringetin 3-*O*-glucoside	C_23_H_24_O_13_	15.73	507.1156	507.1144	2.6
22	Isorhamnetin 3-*O*-hexoside	C_22_H_22_O_12_	15.78	477.1045	477.1038	1.3
23	Isorhamnetin 3-*O*-glucuronide	C_22_H_20_O_13_	16.00	491.0838	491.0831	1.2
24	Dihydrokaempferol 3-*O*-rhamnoside	C_21_H_22_O_10_	16.08	433.1147	433.1140	1.6

**Table 2:** Anthocyanin M^+•^ ions identified in Clinton grape extract. Identification scores calculated with respect to the theoretical mass are reported. Numbers correspond to the peaks of chromatogram in Figure 4.

Peak	Anthocyanins	Formula	Rt (min)	M^+•^	Error (ppm)
				Experimental	
				*m*/*z*	
1	Cyanidin-3,5-*O*-diglucoside	C_27_H_31_O_16_	11.24	611.1612	1.1
2	Petunidin-3,5-*O*-diglucoside	C_28_H_33_O_17_	11.52	641.1718	1.0
3	Delphinidin-3-*O*-monoglucoside	C_21_H_21_O_12_	12.03	465.1033	4.0
4	Peonidin-3,5-*O*-diglucoside	C_28_H_33_O_16_	12.14	625.1769	1.1
5	Delphinidin acetyldiglucoside	C_29_H_33_O_18_	12.16	669.1667	−0.1
6	Malvidin-3,5-*O*-diglucoside	C_29_H_35_O_17_	12.24	655.1874	1.2
7	Cyanidin-3-*O*-monoglucoside	C_21_H_21_O_11_	12.57	449.1084	1.2
8	Petunidin-3-*O*-monoglucoside	C_22_H_23_O_12_	12.74	479.1190	1.7
9	Peonidin-3-*O*-monoglucoside	C_22_H_23_O_11_	13.12	463.1240	−2.0
10	Malvidin-3-*O*-monoglucoside	C_23_H_25_O_12_	13.27	493.1346	1.6
11	Delphinidin-3-*O*-acetylmonoglucoside	C_23_H_23_O_13_	13.59	507.1139	0.8
12	Petunidin caffeoyl diglucoside	C_37_H_39_O_20_	13.87	803.2035	−2.2
13	Delphinidin *p*-coumaroyldiglucoside	C_36_H_37_O_19_	13.91	773.1929	1.1
14	Petunidin-3-*O*-acetylmonoglucoside	C_24_H_25_O_13_	14.14	521.1295	1.1
15	Delphinidin-3-*O*-caffeoylmonoglucoside	C_30_H_27_O_15_	14.18	627.1350	1.3
16	Petunidin *p*-coumaryldiglucoside	C_37_H_39_O_19_	14.36	787.2086	1.1
17	Cyanidin *p*-coumaryldiglucoside	C_36_H_37_O_18_	14.36	757.1980	1.0
18	Malvidin-3-*O*-acetylmonoglucoside	C_25_H_27_O_13_	14.64	535.1452	1.8
19	Peonidin-3-*O*-acetylmonoglucoside	C_24_H_25_O_12_	14.65	505.1346	−1.6
20	Petunidin-3-*O*-caffeoylmonoglucoside	C_31_H_29_O_15_	14.65	641.1506	1.8
21	Malvidin *p*-coumaryldiglucoside	C_38_H_41_O_19_	14.60	801.2242	−0.8
22	Delphinidin-3-*O*-*p*-coumarylmonoglucoside	C_30_H_27_O_14_	14.82	611.1401	2.0
23	Peonidin *p*-coumaryldiglucoside	C_37_H_39_O_18_	14.85	771.2136	−0.8
24	Malvidin-3-*O*-caffeoylmonoglucoside	C_31_H_31_O_15_	15.14	655.1663	−3.2
25	Cyanidin-3-*O*-*p*-coumarylmonoglucoside	C_30_H_27_O_13_	15.24	595.1452	1.0
26	Petunidin-3-*O*-*p*-coumarylmonoglucoside	C_31_H_29_O_14_	15.30	625.1557	1.1
27	Malvidin-3-*O*-*p*-coumarylmonoglucoside	C_32_H_31_O_14_	15.77	639.1714	1.3
28	Peonidin-3-*O*-*p*-coumarylmonoglucoside	C_31_H_29_O_13_	15.78	609.1608	0.2

**Table 3:** Stilbene [M-H]^−^ ions identified in Primitivo grape extract. Identification scores calculated with respect to the theoretical mass are reported. Numbers correspond to the peaks of chromatogram in Figure 6.

Peak	Stilbene compounds	Formula	Rt (min)	[M-H]^−^		Error (ppm)
				Experimental	Theoretical	
				*m*/*z*	*m*/*z*	
1	*E*-Astringin (piceatannol glucoside)	C_20_H_21_O_9_	13.94	405.1198	405.1191	1.7
2	*E*-Piceid	C_20_H_21_O_8_	14.94	389.1249	389.1242	1.8
3	*Z*-Astringin	C_20_H_21_O_9_	15.12	405.1190	405.1191	−0.2
4	Piceatannol	C_14_H_11_O_4_	15.92	243.0666	243.0663	1.2
5	*Z*-Piceid	C_20_H_21_O_8_	15.97	389.1244	389.1242	0.5
6	Dimer 1 (pallidol)	C_28_H_21_O_6_	16.50	453.1347	453.1344	0.7
7	Pallidol-3-*O*-glucoside	C_34_H_31_O_11_	17.01	615.1869	615.1872	−0.5
8	Dimer 2	C_28_H_21_O_6_	17.16	453.1345	453.1344	0.2
9	*trans*-Resveratrol	C_14_H_11_O_3_	17.29	227.0716	227.0714	0.9
10	Caraphenol B	C_28_H_21_O_7_	17.91	469.1292	469.1293	−0.2
11	Tetramer 1	C_56_H_41_O_12_	18.10	905.2602	905.2604	−0.2
12	Tetramer 2	C_56_H_41_O_12_	18.49	905.2607	905.2604	0.3
13	Dimer 3 (*Z*-*ε*-viniferin)	C_28_H_21_O_6_	18.61	453.1346	453.1344	0.4
14	Dimer 4 (*E*-*ε*-viniferin)	C_28_H_21_O_6_	18.81	453.1346	453.1344	0.4
15	Trimer 1 (*Z*-miyabenol C)	C_42_H_31_O_9_	18.84	679.1976	679.1974	0.3
16	Trimer 2 (*E*-miyabenol C)	C_42_H_31_O_9_	18.93	679.1977	679.1974	0.4
17	Dimer 5 (*δ*-viniferin)	C_28_H_21_O_6_	19.56	453.1342	453.1344	−0.4
